# Low Maternal Microbiota Sharing across Gut, Breast Milk and Vagina, as Revealed by 16S rRNA Gene and Reduced Metagenomic Sequencing

**DOI:** 10.3390/genes9050231

**Published:** 2018-05-01

**Authors:** Ekaterina Avershina, Inga Leena Angell, Melanie Simpson, Ola Storrø, Torbjørn Øien, Roar Johnsen, Knut Rudi

**Affiliations:** 1 Department of Chemistry, Biotechnology and Food Science, University of Life Sciences, 1430 Ås, Norway; inga.angell@nmbu.no (I.L.A.); knut.rudi@nmbu.no (K.R.); 2 Department of Public Health and Nursing, Norwegian University of Science and Technology, NTNU, 7491 Trondheim, Norway; melanie.simpson@ntnu.no (M.S.); ola.storro@ntnu.no (O.S.); torbjorn.oien@ntnu.no (T.Ø.); roar.johnsen@ntnu.no (R.J.)

**Keywords:** maternal microbiota, sequencing, reduced metagenomics

## Abstract

The maternal microbiota plays an important role in infant gut colonization. In this work we have investigated which bacterial species are shared across the breast milk, vaginal and stool microbiotas of 109 women shortly before and after giving birth using 16S rRNA gene sequencing and a novel reduced metagenomic sequencing (RMS) approach in a subgroup of 16 women. All the species predicted by the 16S rRNA gene sequencing were also detected by RMS analysis and there was good correspondence between their relative abundances estimated by both approaches. Both approaches also demonstrate a low level of maternal microbiota sharing across the population and RMS analysis identified only two species common to most women and in all sample types (*Bifidobacterium longum* and *Enterococcus faecalis*). Breast milk was the only sample type that had significantly higher intra- than inter- individual similarity towards both vaginal and stool samples. We also searched our RMS dataset against an in silico generated reference database derived from bacterial isolates in the Human Microbiome Project. The use of this reference-based search enabled further separation of *Bifidobacterium longum* into *Bifidobacterium longum* ssp. *longum* and *Bifidobacterium longum* ssp. *infantis*. We also detected the *Lactobacillus rhamnosus* GG strain, which was used as a probiotic supplement by some women, demonstrating the potential of RMS approach for deeper taxonomic delineation and estimation.

## 1. Introduction

Microorganisms that colonize humans are tightly associated with our health and well-being. Our gut microbial community, for example, plays a role in immune system development and response to medications [1,2]. Its composition and structure is also associated with a number of mental and digestive disorders [3,4]. Numerous studies on germ-free animals also suggest the importance of microbes in the proper functioning of the host [5]. This evidence has led to a recently-developed view of humans as holobionts—as “super-organisms”, where both the host and its microbiome are so closely interconnected that they become inseparable [6,7]. However, some researchers argue that not all microbes that thrive in our bodies should be regarded as essential parts of the super-organism since some of them are merely passersby that were acquired “by chance” [8]. They point out that for any microbe to be viewed as an essential part of a super-organism it should be vertically transmitted and present in a large proportion of the population, in addition to having a symbiotic relationship with the host. Indeed, a mothers microbiome plays an important role in the shaping of her infant’s gut microbiota. Vaginally born children are initially colonized by their mother’s vaginal microbes and this imprinting can be seen for years [9]. There are also reports of maternal gut microbes that have been detected in the gut microbiota of their offspring [10], but whether this colonization happens pre- or postnatally is still debated [11]. Breast milk also contributes to the initial structuring of the infant gut microbiota and its composition over several years [12,13]. In addition to direct transmission of viable bacteria from mother to child, breast milk contains maternal IgA that reflects the mother’s own microbial exposures and which may prime the infant immune system [14].

There are two major approaches for microbial community analysis—targeted marker gene sequencing or whole genome shotgun (metagenome) sequencing. Targeted sequencing uses only a small fraction of the genome from each microbial community member and is often insufficient for species separation [15]. On the other hand, metagenome sequencing provides deeper taxonomic resolution by utilizing information from a large proportion of each genome, yet requires substantial computational power to reconstruct the genomes [16,17]. Moreover, this approach results in the sequencing of a number of DNA fragments that are excessive to the tasks of species resolution and functional assessment and thus sequencing depth is lost without providing new information. A reduced metagenomic sequencing (RMS) approach combines the advantages of both targeted and shotgun metagenomics. Depth of sequencing is increased by “targeting” special signatures whilst resolution depth is also increased by sequencing more than one signature from each microbial community member. Sequencing of double digest restriction site associated DNA (ddRAD) fragments was recently assessed for this purpose [18]. Briefly, the ddRAD approach uses a mixture of two restriction enzymes, N1aIII and HpyCH4IV, to digest template DNA prior to attachment of adapters to fragments for PCR amplification and sequencing. Here we use an alternative approach that was widely used for species differentiation in the past, namely amplicon length polymorphism (AFLP) [19], coupled with deep sequencing. The concept is similar to that of the ddRAD method, but EcoRI and MseI restriction enzymes are used for DNA digestion. We have previously demonstrated successful use of this method combined with the MiSeq Illumina platform for metagenome estimation and functional assessment [20].

In this work, we aimed to identify bacterial species that (a) were shared across breast milk, vaginal swab and stool samples of 109 mothers from mid-Norway and (b) were present in a large proportion of this population. We hypothesized that bacteria fulfilling these two criteria are more likely to constitute a holobiotic part of the human microbiota.

## 2. Materials and Methods

### 2.1. Study Cohort

All samples analyzed in the current work were collected during the previously published Probiotics in Prevention of Allergy among Children in Trondheim (ProPACT) study [21]. Participants were asked to collect stool samples during pregnancy and 3 months after birth and breast milk samples at 10 days and 3 months after birth. Both stool and breast milk samples were to be frozen immediately at −20 °C and delivered to the laboratory within 4 weeks for further storage at −80 °C until analysis. Vaginal swab samples were collected either by the participants 4 weeks prior to their expected due date and immediately placed into the home freezer until they were sent for further storage at −80 °C, or by medical staff at admission to the hospital for labor.

We have previously described microbial composition of the breast milk [22], vaginal swab [23] and stool [24] samples independently of each other using 16S rRNA gene sequencing. In this work, we sought to analyze the complete maternal microbiome and identify bacteria shared between the breast milk, vaginal swab and stool samples. Therefore, we have combined and compared the three individually sequenced datasets, keeping only individuals who had information for all three sample types. The final 16S rRNA dataset comprised of 327 samples (109 breast milk; 109 vaginal swab and 109 stool samples) from 109 women (Table S1). For some women, data was available from two collection time points for either breast milk, stool or vaginal swabs samples. In these cases, the 16S rRNA results were averaged across the two time points since the primary aim in this work was to identify microbes shared across maternal body sites around the time of birth. The ProPACT study is a randomized, placebo controlled trial, in which women who were allocated to the probiotic arm of the trial received a fermented milk product which contained *Lactobacillus rhamnosus* GG (LGG), *Bifidobacterium animalis* ssp. *lactis* Bb-12 and *Lactobacillus acidophilus* La-5 from 36 weeks gestation until 3 months after birth. Based on our previous analyses, probiotic intake did not affect the overall microbiota of these samples [22,23,24]. Therefore, we have not stratified mothers into probiotic/placebo groups in this work. The information on probiotic intake is available in Table S1 and the presence of the administered bacteria was assessed in the RMS data.

### 2.2. Generation of the Common 16S rRNA Dataset

16S rRNA gene amplicon datasets for each sample type were generated separately and at different time points. To create a common dataset, we combined OTUs that shared more than 97% identity with each other, into OTU sets. Each OTU set comprised of one representative OTU sequence from the breast milk (B), vaginal swab (V) and stool (S) datasets respectively and therefore we called these sets BVS-OTUs. We then filtered BVS-OTUs based on their taxonomy assignments, discarding any BVS-OTUs that included representative sequences with two conflicting assignments at the same taxonomy level. For those BVS-OTUs that had representative sequences classified at different taxonomy levels, the lowest assigned level was kept for identification.

### 2.3. Generation of the RMS Dataset

A subset of 34 samples from 16 women was subjected to RMS analysis. Samples were selected from women with high counts of OTUs that were included in the BVS-OTUs in at least two sample types. As such the RMS analysis included 13 of the 109 women in the combined 16S rRNA gene sequencing analysis and was augmented with another 3 women who also had high levels of these OTUs in two sample types, but where their third sample type was not available (Table S3). Previously isolated DNA (~10 ng) was digested using 8 U of EcoRI (New England Biolabs, UK) and 4 U of MseI (New England Biolabs, UK) with addition of 1× CutSmart Buffer (New England Biolabs, UK) at 37 °C for 1 h and AFLP adapters (EcoRI-F: 5′-CTCGTAGACTGCGTACC-3′; EcoRI-R: 5′-AATTGGTACGCAGTCTAC-3′; MseI-F: 5′-GACGATGAGTCCTGAG-3′; MseI-R: 5′-TACTCAGGACTCAT-3′) were ligated. Resulting fragments were enriched in PCR reaction with EcoRI (5′-GACTGCGTACCAATTC-3′) and MseI (5′-GATGAGTCCTGAGTAA-3′) primers [19]. Cycling protocol comprised of polymerase activation step of 95 °C for 15 min followed by 25 cycles of denaturation at 95 °C for 30 s, annealing at 56 °C for 1 min and elongation at 72 °C for 30 s with a final elongation step at 72 °C for 7 min.

Fragments of around 500 bp were selected using AmPure XP beads (Beckman Coulter, Brea, CA, USA), quantified, normalized and then sequenced on HiSeq platform (Illumina, San Diego, CA, USA) at the Norwegian Sequencing Centre (Oslo, Norway).

Sequencing reads (104 bp) were filtered against the human database using BBMap [25] and then additionally BLAST-searched and filtered against the NCBI Human genome reference database. The remaining reads were clustered at 97% identity level using USEARCH v 8.0 [26].

RMS analysis was verified using mock communities of four bacteria mixed in various proportions including one negative control with no genomic DNA template (Appendix A).

### 2.4. Generation of the RMS Database Based on the Human Microbiome Project Representative Isolates (HMP-RMS Database)

Reference genomes of bacteria isolated from oral, gastrointestinal and urogenital tracts, were downloaded from the Microbial Reference Genomes database (Human Microbiome Project; HMP). For each of the genomes, a restriction map with EcoRI and MseI enzymes was created using MATLAB R2016b (MathWork Inc., Natick, MA, USA); and those fragments that contained a combination of EcoRI/MseI or MseI/EcoRI cutting sites on their ends, were selected using MATLAB R2016b (MathWork Inc., Natick, MA, USA). All fragments were pooled in one database and clustered on a 97% identity level using USEARCH v 8.0 [26] restricting minimal cluster size to one (-*minsize* 1). The complete HMP-RMS database comprised of 707,811 clusters from 970 HMP reference isolates belonging to 475 bacterial species.

### 2.5. Statistical Analyses

All analyses were performed in MATLAB R2016b (MathWorks Inc., Natick, MA, USA) unless stated otherwise. Similarity between samples from different body sites was assessed using Jaccard distance. To compensate for different sequencing depths, a cutoff of 1% was set as a detection threshold for diversity estimation. This cutoff ensured that any bacterial community with a true relative amount over 2% would exceed the detection threshold, even in the most-shallow sequenced dataset which was rarefied to 1000 sequences per sample (Figure S1).

Non-parametric Kruskal-Wallis test with Benjamini-Hochberg FDR correction for multiple testing was used for assessment of statistical significance at the 5% level.

### 2.6. Data Availability

All generated datasets can be made available upon request to the corresponding author.

## 3. Results

### 3.1. Sharing of Bacteria between Breast Milk, Vaginal and Stool Microbiota, as Determined by 16S rRNA Gene Sequencing

In total, 307, 463 and 1313 OTUs were detected in the breast milk, vaginal swabs and stool datasets respectively. Among those, we detected 67 BVS-OTUs (i.e., sets of OTUs from breast milk, vaginal swab and stool datasets that have ≥97% similarity with each other). The majority of these BVS-OTUs belonged to Bacilli and Clostridia classes and their cumulative relative abundance comprised 72% of the total microbial load in all breast milk samples, 45% in vaginal swab samples and 15% in stool samples (Figure S2).

Generally, there was no similarity between the overall microbial communities detected in the three samples types when they were compared between individuals. Within individuals, comparison across samples types also indicated that these sample types have no similarity for most women. However, the Jaccard dissimilarity score for a few women indicated at least some similarity with sharing of several BVS-OTUs across sample types, particularly between breast milk and stool samples (*n* = 9) and between breast milk and vaginal swabs samples (*n* = 5) (Figure S3).

None of BVS-OTUs were detected in more than half of the tested population (at least 55 women) in all three sample types (i.e., prevalence > 50% in the breast milk, vaginal swab and stool datasets). We then searched for BVS-OTUs that were shared across all three sample types within a given individual. Thirty-four women (31% of the study cohort) were found to have at least one common BVS-OTU across all three sample types. In most cases, each woman had only one common BVS-OTU among her samples (Table S2) with BVS-OTU identified as *Streptococcus* being the most frequently encountered individually shared (found in 15 out of 109 women; 13.2% prevalence).

### 3.2. RMS Analysis of Samples from Mothers with Shared BVS-OTUs

We generated 28,657,029 raw human DNA-free RMS reads (Table S4) for 34 samples from 16 women and clustered these raw RMS reads with 97% similarity threshold, resulting in 1,232,959 RMS clusters. Whilst the majority of raw RMS reads belonged to stool samples (67.4% of the sequencing data), a similar number of RMS clusters were detected in the different sample types (426,892, 373,230 and 432,837 RMS clusters for breast milk, vaginal swabs and stool samples, respectively). The breast milk and vaginal swab samples exhibited the greatest similarity, with 23,775 RMS clusters found in common in at least half of the tested population, as compared to 220 and 107 RMS clusters shared between breast milk/stool samples and stool/vaginal swab samples, respectively.

**Figure 1:**
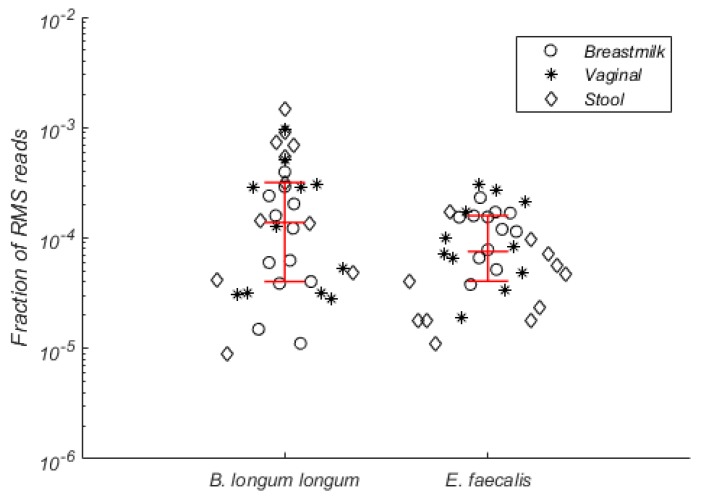
Fraction of RMS reads that belong to commonly detected RMS clusters identified as *B. longum* ssp. *longum* and *E. faecalis.* Median values, as well as 25th and 75th percentile, are depicted in red.

To address inter-individual sharing of bacteria, we searched for RMS clusters that were detected in both breast milk, vaginal swabs and stool samples of more than half of tested women (*n* > 8). In total, we identified eighty-six RMS clusters that satisfied the criteria. Based on BLAST searches of these RMS clusters against the NCBI nucleotide database (Table S5), most of them belonged to *Bifidobacterium longum* ssp. *longum* and *Enterococcus faecalis* (31 and 28 common RMS clusters respectively). Collectively, these clusters were detected in all tested samples (*n* = 34) and represented around 10^−4^ fraction of all clustered RMS reads (Figure 1).

We then searched for RMS clusters shared among different sample types within each individual separately. Each woman shared 1925 (median value; interquartile range IQR = 13,281) RMS clusters among her samples. BLAST search of these clusters against the NCBI database revealed that both *B. longum* ssp. *longum* and *E. faecalis* RMS clusters were also two of the most frequently identified shared clusters within individuals (Table S6). One woman had RMS data on all three sample types. In total, 328,114 RMS clusters were detected in her samples and 405 were shared across all of them. The majority of these shared RMS clusters were identified as *E. coli* and *E. faecalis* (Figure S4). These RMS clusters spanned the whole genomes with an average identity of 97.7% and 96.2% for *E. coli* and *E. faecalis*, respectively and their relative abundance ranged between 0.01% and 0.52%.

### 3.3. Microbiota Sharing between the Study Cohort and the HMP Database

We extracted the genome information for all bacterial isolates obtained from skin, oral and gastrointestinal tracts of the USA residents in the Human Microbiome Project Genome database (downloaded in February 2017), generated RMS clusters from these data (HMP-RMS database) and mapped our raw reads against this database. Twenty-three percent of raw RMS reads from our cohort mapped towards 78,822 in silico generated HMP-RMS clusters, which constituted 11.1% of the whole HMP-RMS database. These clusters belonged to 332 bacterial isolates.

**Figure 2:**
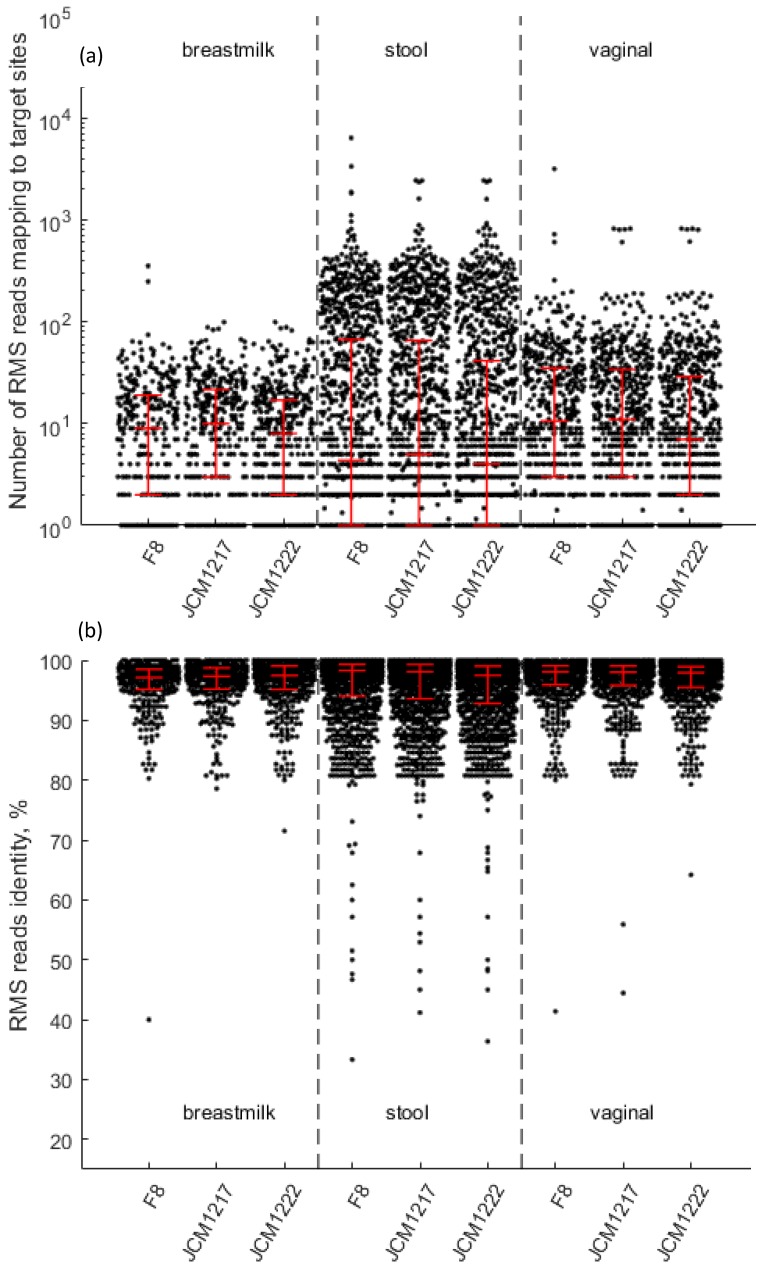
Mapping of raw RMS reads from breast milk, stool and vaginal swab pools towards whole genome sequences of *B. longum*. F8 and JCM1217—*B. longum* ssp. *longum*; JCM1222—*B. longum* ssp. *infantis*. Median values, as well as 25th and 75th percentile, are depicted in red; (**a**) Coverage of mapped genome regions; (**b**) Pairwise identity of raw RMS reads towards mapped genome regions.

Forty-two bacterial species from the HMP database were detected in more than 50% of the study cohort in all three sample types (Table S7). *B. longum* reads mapping towards HMP-RMS clusters belonging to *B. longum* ssp. *longum* and *B. longum* ssp. *infantis* were detected in all individuals and all sample types. We therefore pooled raw RMS reads into stool, breast milk and vaginal pools and mapped them onto the genomes of *B. longum* ssp. *longum* and *B. longum* ssp. *infantis*. Two of *B. longum* ssp. *longum* HMP isolates (F8 and JCM1217), as well as *B. longum* ssp. *infantis* JCM1222, had whole genome sequences available in the HMP database of reference genomes. Our mapped reads spanned the whole genomes of each of these isolates with an average coverage ranging from 12.7 to 83.7 reads per mapped genome region and an average identity ranging from 95.3% to 96.7% (Figure 2).

### 3.4. Correlation between RMS and 16S rRNA Analysis

Among the samples subjected to RMS analysis, the high-count BVS-OTUs included species from the *Streptococcus*, *Veillonella*, *Bifidobacterium*, *Blautia*, *Sphingomonas, Anaerococcus, Moraxella* and *Dolosigranulum* genera, as well as some bacterial groups classified down to class level (Table S3).

We mapped raw RMS reads from samples targeting species-classified BVS-OTUs towards the representative sequences of the target species (only those species that either had a whole genome or a scaffold > 1.5 Mbp deposited at the NCBI Genome database). Reads mappings towards the in silico predicted RMS regions of the respective target species were detected in all tested samples (Table S8).

**Figure 3:**
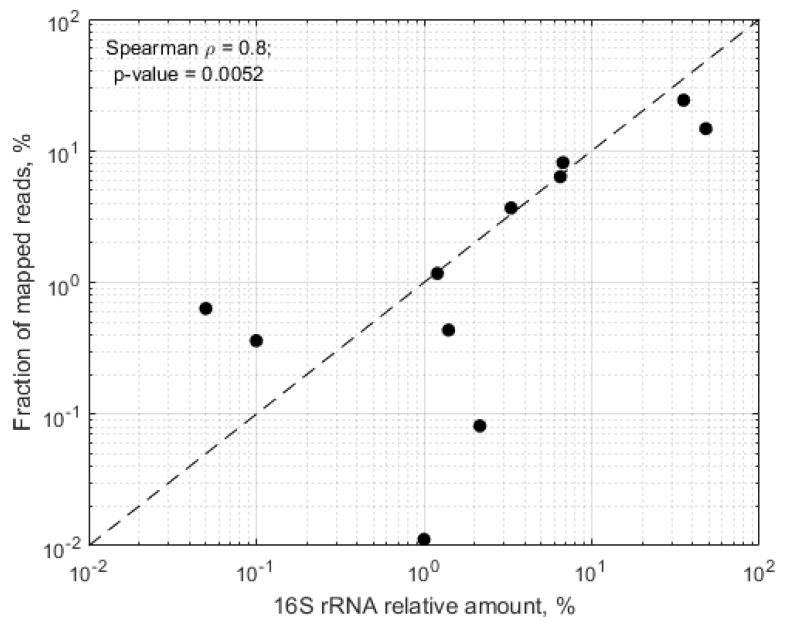
Correlation between 16S rRNA relative abundance and RMS estimates of expected target species in samples where >50% of the mapped regions corresponded to the in silico predicted RMS fragments.

In samples, where more than 50% of the mapped reads mapped towards the in silico predicted RMS regions, we observed significant correlation between the expected relative abundance of the target species and the proportion of raw RMS reads mapping towards the target genomes (Spearman rho = 0.8, *p* = 0.0052) (Figure 3).

Since *Streptococcus* was detected as the most commonly shared BVS-OTU, we extracted raw RMS reads from breast milk and vaginal swab samples from one woman who was predicted to have high relative counts of *Streptococcus* BVS-OTU (>1%). We then mapped these reads against 23 streptococcal species that had whole genome sequences deposited in NCBI Genome database (Table S9). All samples had reads mapping towards streptococci, however genomes of *S. agalactiae* and *Enterococcus saccharolyticus* (previously identified as *S. saccharolyticus*) were most widely represented (Figure S5) across the samples.

*B. longum* and *E. faecalis* were identified as the most commonly detected bacteria across all sample types by the RMS approach, but not through 16S rRNA gene sequencing. Therefore, we returned to the original breast milk, vaginal swab and stool datasets to investigate whether OTUs with >97% similarity towards the 16S rRNA gene of *E. faecalis* V583 and *B. longum* (both subspecies) were detected in the subset of samples subjected to RMS analysis. *B. longum* OTU was detected in nine vaginal swab samples, one breast milk and none of the stool samples. *E. faecalis* OTU was detected in three breast milk, three vaginal swab and two stool samples. To assess whether these low detection rates were a result of rarefaction, we simulated datasets to estimate the probability of detecting a single read from a taxon at varying relative abundances and using rarefication cutoffs of 1000, 2000 and 3000 sequences per sample, as was the case for the breast milk, stool and vaginal swab datasets, respectively. According to these calculations, a taxon that comprises around 0.01% of the community, has a 7% chance of inclusion into the rarefied dataset when limited to 1,000 sequences per sample and this rises to 27% when 3000 sequences per sample is the rarefication cutoff (Figure S6).

### 3.5. Assessment of the DNA Kitome

A control sample with no gDNA template was also included into the mock community analysis in order to assess technical contamination that might rise from the DNA handling kits. In total, 4676 reads were generated by HiSeq sequencing from this negative control. Given that the 10 ng gDNA mock community samples yielded on average 2,557,913 reads per sample; the gDNA template in the negative control probably comprised around 0.02 ng. We BLAST searched all the negative control reads against the Reference Prokaryotic (RefProk) NCBI database (Table S10).

Less than 40% of the reads (1790 of 4676 reads) could be matched against the reference database, using a lenient requirement of at least 50% concordance of the read length. In total, 736 and 47 reads were assigned to *E. faecalis* and *B. longum*, respectively. Since both of these bacteria were detected as the most commonly shared bacteria in maternal samples, we aligned all the negative control reads assigned to these bacteria to all RMS fragment clusters that were shared among maternal samples. The best-matching pairwise alignments between negative control reads and fragment clusters had 36.4 ± 9.8% and 61.0 ± 29.0% identity for *E. faecalis* and *B. longum* reads, respectively. Seven of *B. longum* negative control reads were completely identical to RMS fragments shared among maternal samples, whereas none of *E. faecalis* negative control reads matched maternal fragment clusters. Moreover, all of the negative control reads matched uniquely towards *E. faecalis* V583 which was included into the mock community. RMS clusters from maternal samples, however, gave best BLAST hits against numerous *E. faecalis* strains (KB1, L12, AMB05, CLB21560, D32, LD33) in addition to V583.

### 3.6. Search for Probiotic Bacteria in RMS Dataset

As described, the samples were collected from women participating in a randomised, placebo controlled trial and 10 out of the 16 women in the RMS dataset had received a probiotic supplement containing *Lactobacillus rhamnosus* GG (LGG), *Bifidobacterium animalis* ssp. *lactis* Bb-12 and *Lactobacillus acidophilus* La-5. We have searched for RMS clusters assigned to these bacterial species using BLAST against the Reference Prokaryotic (RefProk) NCBI database. All of the probiotic species were identified in the dataset (Table S12) with 100% identity over the whole read length (104 bp). *B. animalis* ssp. *lactis* was detected only in one stool sample; *L. acidophilus*—in two breast milk samples, two vaginal swabs and one stool samples, whereas *L. rhamnosus* was found in one vaginal swab and four stool samples (Table S13). Interestingly, all of the RMS fragments that were identified as *L. rhamnosus*, were mapped specifically to LGG although there are 106 different *L. rhamnosus* strains in the RefProk NCBI database.

## 4. Discussion

There are number of papers addressing sharing of bacteria across various body sites [27,28], but none have focused on the sharing of the maternal microbiota. Initially, we compared microbiotas of 109 women using 16S rRNA gene sequencing data. BVS-OTUs belonging to the Clostridia class were highly represented in stool samples and remained low in breast milk and vaginal swabs, whereas those belonging to the Bacilli class accounted for the majority of the shared microbiota in the latter two sample types. Interestingly, over 70% of the total microbial load across all breast milk samples can be attributed to one or more of the 67 BVS-OTUs identified. Moreover, breast milk was the only sample type that was significantly more similar to either vaginal swabs or stool samples from the same mother than among different mothers. We believe that this observation may point towards translocation of maternal bacteria to breast milk. Several reports have shown recovery of intestinal bacteria in breast milk [13,29,30] and an internal entero-mammary pathway has been proposed to explain this translocation (reviewed in [31]). This, however, does not explain the individual similarity between vaginal and breast milk samples. Alternatively, the external pathway via hands and skin may also contribute to the aforementioned similarities.

Generally, RMS data corresponded well to 16S rRNA gene sequence data. All the species that were predicted to be found in the samples by 16S rRNA gene sequencing were also detected using the RMS approach. In all cases, reads mapped towards several regions of the target genome and the majority of these regions were covered by more than one RMS read. There was also a significant correlation in the relative abundance estimates between the two approaches. However, there were also some discrepancies between the two datasets. Whilst *Streptococcus* was the most commonly shared bacterium between breast milk, vaginal swabs and stool samples in the 16S rRNA gene sequencing dataset, it was only detected in all sample types for 13.2% of women and none of BVS-OTUs was universally distributed across the study cohort. The RMS approach and deeper HiSeq sequencing also suggested that few bacteria were common among women and body sites, although here it was *B. longum* and *E. faecalis* that were identified as common across the maternal microbiotas in the majority of the women. Both of these species were detected at very low levels, representing around 10^−4^ of the total microbial load. The probability of detecting such low abundant taxa would not have exceeded 30% in the rarefied datasets used for the 16S rRNA gene sequencing analysis and this may explain why 16S rRNA gene sequencing did not identify the sharing of these taxa. Nonetheless, we also investigated the possibility that the recovery of these species was due to contamination through an assessment of the negative control in the mock community analyses. Both *B. longum* and *E. faecalis* were detected in the negative control sample, yet the vast majority of reads in the negative control could not be mapped to the fragment clusters from the maternal samples. Reassuringly, this suggests that the presence of *B. longum* and *E. faecalis* in all samples in the RMS dataset is not artefactual. Indeed, they are common residents of breast milk, gut and vaginal microbiota [32,33,34] and both of them are capable of being transmitted from mother to child [13].

*B. longum* consists of three subspecies (*longum*, *infantis* and *suis*), but standard 16S rRNA gene sequencing is usually unable to differentiate between them due to a low degree of variation in 16S rRNA gene [35]. Therefore, other genes are normally targeted in order to distinguish between these subspecies [35,36]. Using an RMS approach, we were able to separate between two subspecies, *B. longum* ssp. *longum* and *B. longum* ssp. *infantis,* demonstrating that RMS may be an efficient alternative to the shotgun metagenomics approach despite the lower complexity of the data. The RMS approach may also offer efficient strain-level resolution. We observed some indication of this potential in the specific identification of the *L. rhamnosus* GG strain, which was used as a probiotic supplement in our dataset. However, this needs to be more thoroughly tested using mock communities with various strains of the same species. 

One mother had RMS information on all three sample types and the majority of RMS clusters shared between all her samples belonged to *E. coli*. *E. coli* is a common resident of the human gut and it has also been detected in breast milk of healthy women [37]. Vaginal *E. coli*, on the other hand, is often associated with a higher risk of urogenital infections and various complications during pregnancy, including premature rupture of membranes [38]. This mother, however, did not report any symptoms of vaginal infections during pregnancy and she gave birth at term.

## 5. Conclusions

In this work we demonstrate a low degree of overall maternal microbiota sharing suggested both by 16S rRNA gene sequencing data on 109 mothers, as well as by deeper reduced metagenomics sequencing of samples from 16 mothers. Interestingly, breast milk was the only sample type with higher intra- than inter-individual similarity, coinciding with higher total bacterial load of shared microbial classes. Based on RMS analysis, only three bacteria, *Bifidobacterium longum* ssp. *longum, Bifidobacterium longum* ssp. *infantis* and *Enterococcus faecalis*, were frequently encountered in all sample types across the tested population. The genomes of these bacteria were evenly covered, although with varying identity levels, suggesting species, but not strain sharing across the population. The wide spread of *B. longum* and *E. faecalis* in breast milk, vaginal and stool samples of mothers, was possibly missed by 16S rRNA gene sequencing due to low rarefaction levels used for data normalization. We also demonstrate that the use of a reduced metagenomics approach for identification of bacterial species can be successfully applied for resolution of bacteria down to a subspecies level while reducing the complexity of the metagenomic dataset.

## Supplementary Materials

The following are available online at http://www.mdpi.com/2073-4425/9/5/231/s1, Figure S1: Probability of OTU to exceed a given detection threshold given its true relative amount; sequencing depth is set to 1000 sequences per sample. Probability of detection above the set threshold is calculated based on 500 random permutations; Figure S2: Relative amount of BVS-OTUs in breast milk, stool and vaginal swab samples (Actinob—Actinobacteria; γ-Prot—Gammaproteobacteria; Bacter—Bacteroidia; β-Pr—Betaproteobacteria; α-—Alphaproteobacteria; F—Fusobacteria; Cyan—Cyanobacteria); Figure S3: Jaccard index of dissimilarity between vaginal, breast milk and stool samples. Jaccard index ranges from 0 (identical) to 1 (completely different) and is calculated based on the proportion of common taxa detected between pairs of samples. To herself: intraindividual dissimilarity; to others: interindividual dissimilarity. Median values, as well as 25th and 75th percentile, are depicted in red; Figure S4: Taxonomic distribution of 405 RMS clusters shared across breastmilk, stool and vaginal swab samples of one individual; Figure S5: Number of regions in *Streptococcus* target genomes that are covered by raw RMS reads from samples of the woman in who *Streptococcus* BVS-OTU was detected based on 16S rRNA data. B1—breast milk sample; V18 and V19—vaginal swab samples; Figure S6: Probability of detecting a single read from a taxon using a given rarefaction level and its true fraction in the community. Probability is calculated based on 500 random permutations; Figure S7: Verification of RMS analysis. (a) Number of RMS reads mapping towards in silico generated RMS clusters from target species. (b) Correlation between “true” and “estimated” target bacteria concentrations; Table S1: Description of the study cohort; Table S2: List of women who shared BVS-OTUs across all three sample types; Table S3: List of samples used for RMS analysis; Table S4: Number of RMS sequencing reads; Table S5: BLAST search of RMS clusters that are shared across all sample types in at least half of the population, against the NCBI genome database; Table S6: *E. faecalis* and *B. longum* shared across breast milk, stool and vaginal swabs of individuals; Table S7: List of HMP bacterial species that RMS reads mapped towards; Table S8: Correlation between 16S rRNA prediction and RMS detection. RMS reads mapping towards target species; Table S9: List of *Streptococcus* species genomes used for mapping of raw RMS reads; downloaded from the NCBI Genome database; Table S10: BLAST search of negative control reads against the NCBI Reference Prokaryotic RefProk database; Table S11: Composition of mock community samples; Table S12: Identification of probiotic bacteria in the RMS dataset; Table S13: List of samples where probiotic bacteria were detected.

## Appendix A

### Verification of the RMS Sequencing Approach

Mock communities of *E. coli* ATCC25922; *E. faecalis* V583; *B. longum* DSM20219 and *B. infantis* DSM20088 mixed in varying proportions ranging from 0% to 100% (Table S11), were used to assess the sensitivity of the AFLP sequencing in prediction and identification of bacterial strains. More than 95% of the AFLP reads that were generated from pure bacterial strains mapped only to the target *in silico* AFLP fragments (Figure S7a). There also was high correlation (R^2^ > 0.93) between true bacterial mixtures and bacterial concentrations estimated based on the number of unique AFLP fragment reads in these mixtures (Figure S7b).
