# Supplementary material for: Low Maternal Microbiota Sharing across Gut, Breast Milk and Vagina, as Revealed by 16S rRNA Gene and Reduced Metagenomic Sequencing
Source: Genes (Basel). 2018 May 1;9(5):231. doi: 10.3390/genes9050231 (PMC5977171; doi:10.3390/genes9050231)
Supplement: Supplementary file 1 [file genes-09-00231-s001.zip › genes-295934 - supplementary figures.pdf]

Supplementary Figures

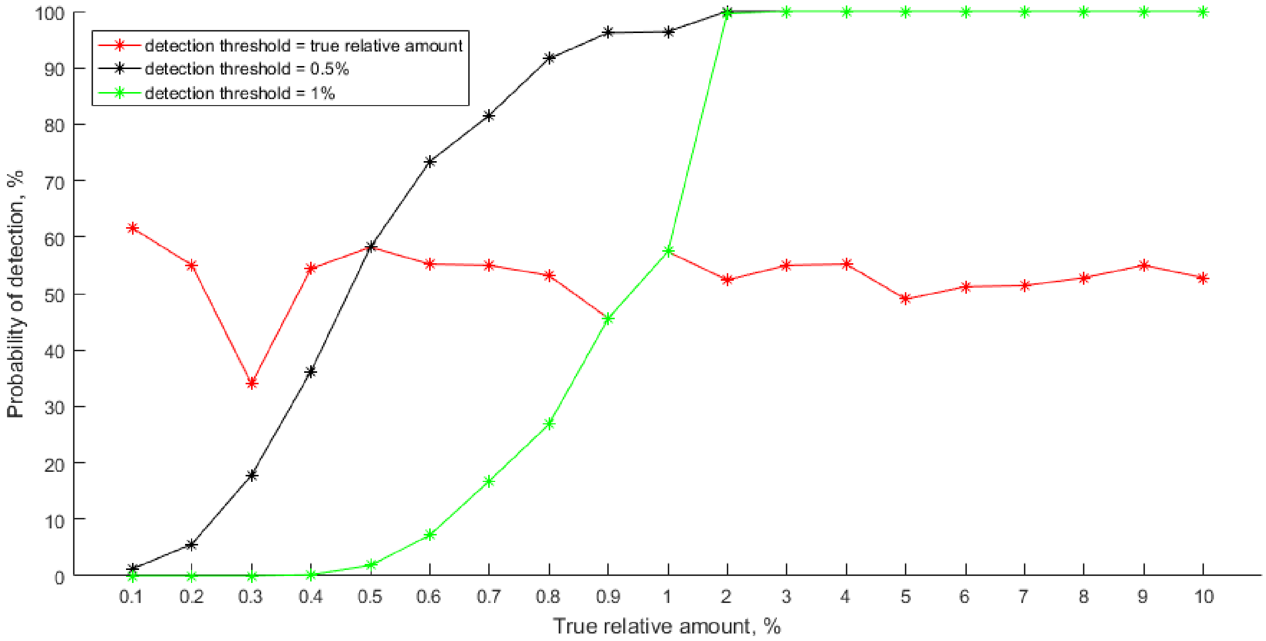

Figure S1. Probability of OTU to exceed a given detection threshold given its true relative amount; sequencing depth is set to 1000 sequences per sample. Probability of detection above the set threshold is calculated based on 500 random permutations.

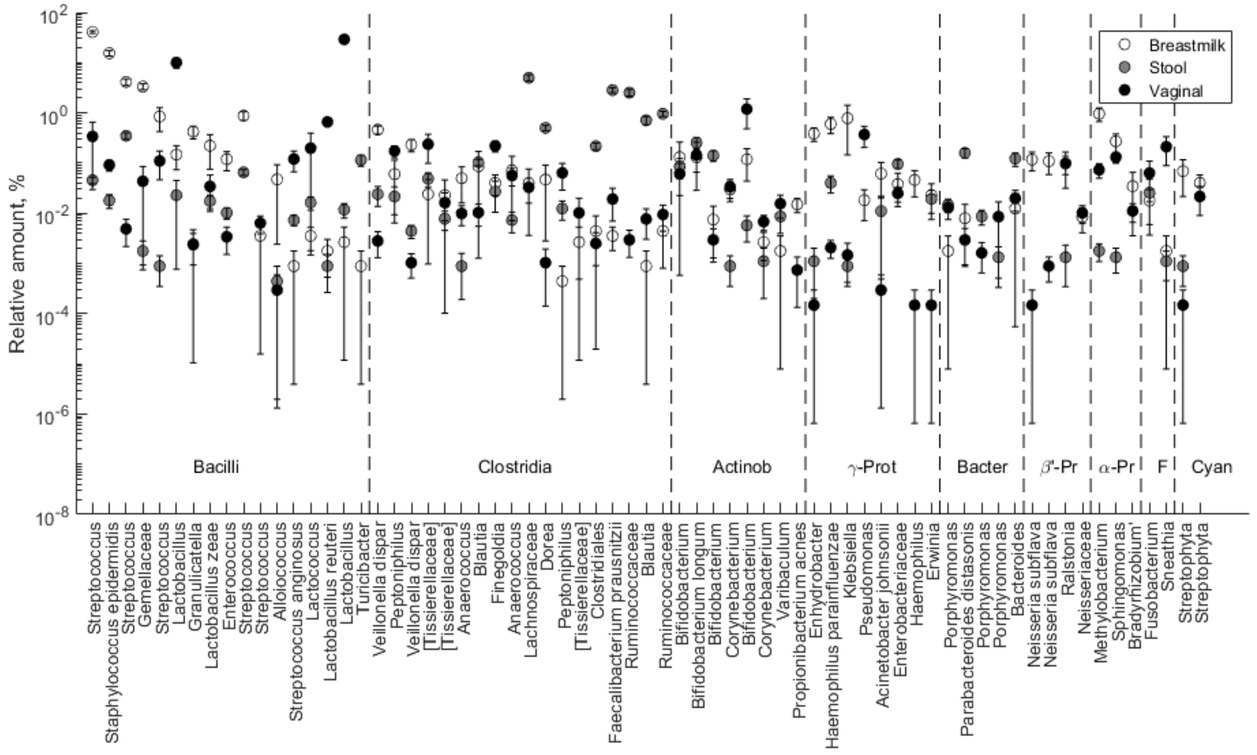

Figure S2. Relative amount of BVS-OTUs in breast milk, stool and vaginal swab samples (Actinob – Actinobacteria; γ-Prot – Gammaproteobacteria; Bacter – Bacteroidia; β-Pr – Betaproteobacteria; α-Pr – Alphaproteobacteria; F – Fusobacteria; Cyan – Cyanobacteria)

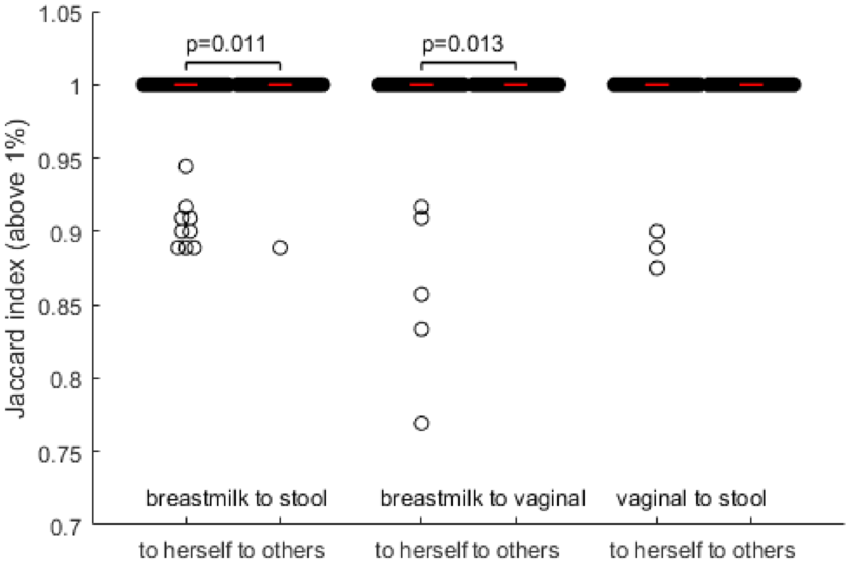

Figure S3. Jaccard index of dissimilarity between vaginal, breastmilk and stool samples. Jaccard index ranges from 0 (identical) to 1 (completely different) and is calculated based on the proportion of common taxa detected between pairs of samples. To herself: intraindividual dissimilarity; to others: interindividual dissimilarity. Median values, as well as 25th and 75th percentile, are depicted in red.

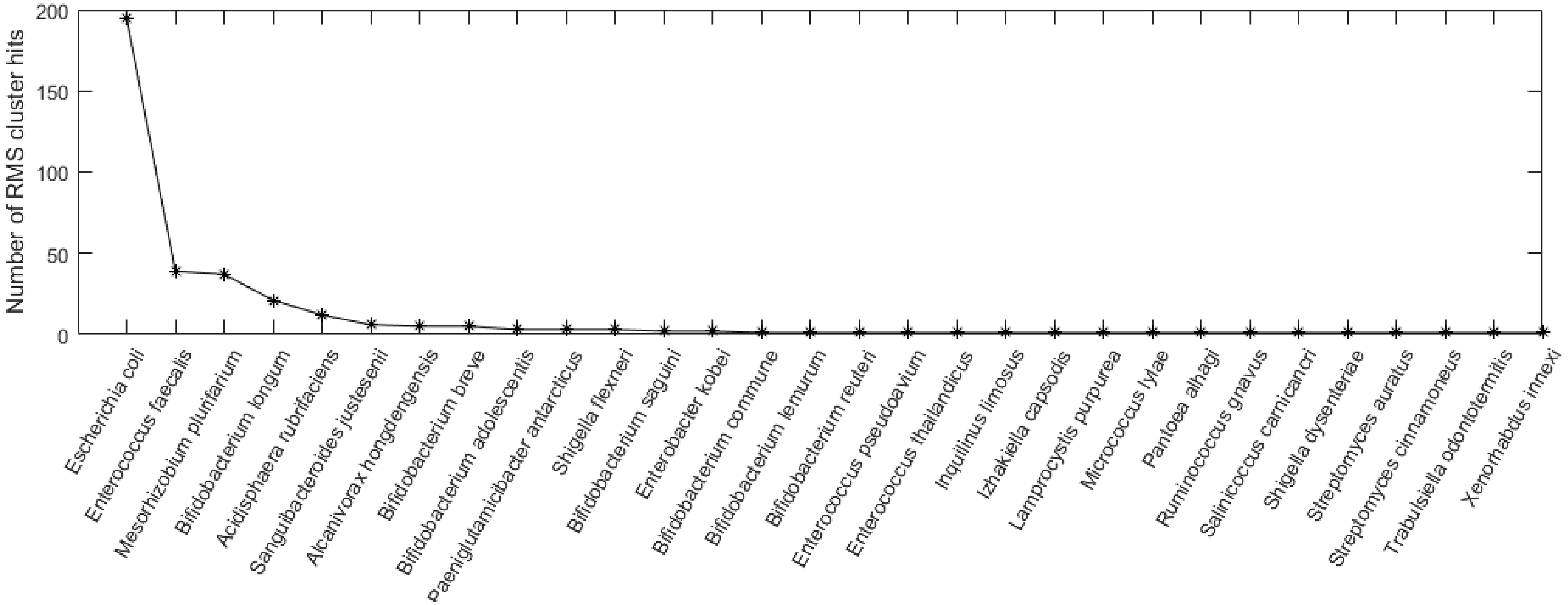

Figure S4. Taxonomic distribution of 405 RMS clusters shared across breastmilk, stool and vaginal swab samples of one individual

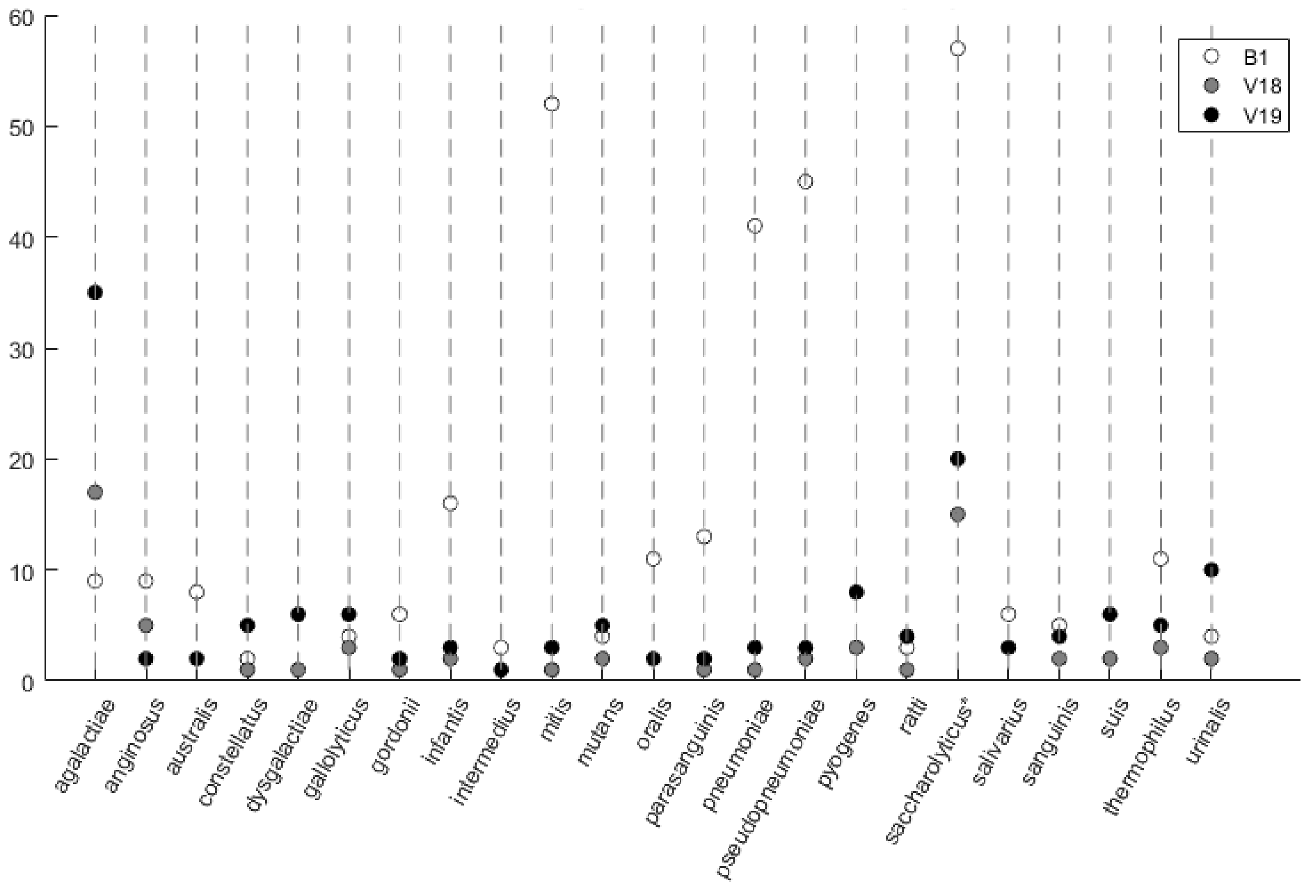

Figure S5. Number of regions in *Streptococcus* target genomes that are covered by raw RMS reads from samples of the woman in who *Streptococcus* BVS-OTU was detected based on the 16S rRNA data. B1 – breastmilk sample; V18 and V19 – vaginal swab samples.

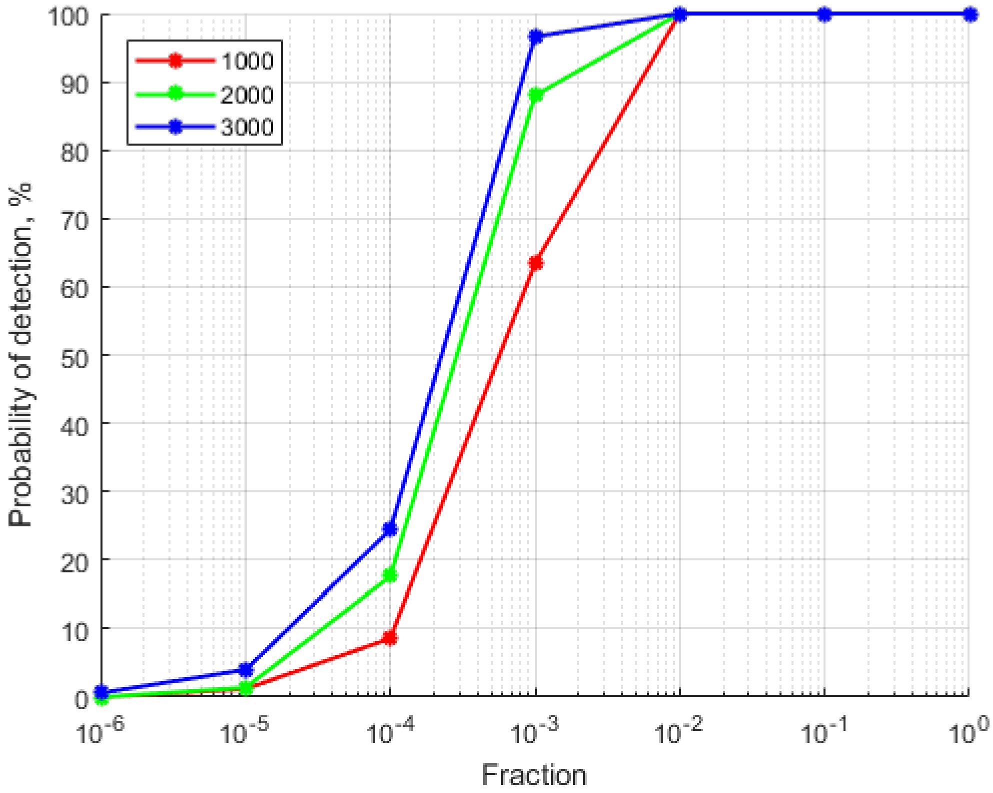

Figure S6. Probability of detecting a single read from a taxon using a given rarefaction level and its true fraction in the community. Probability is calculated based on 500 random permutations.

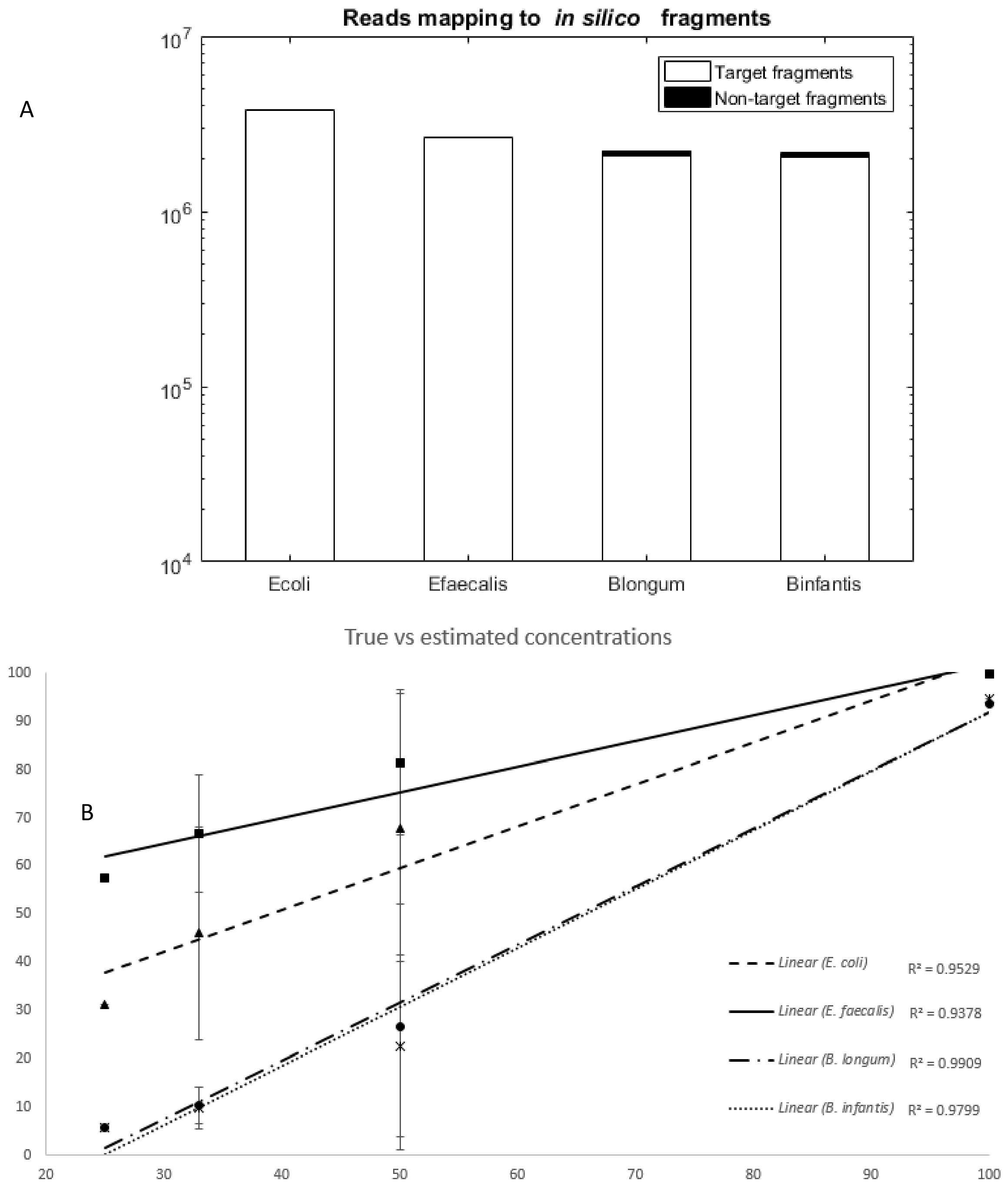

Figure S7. Verification of RMS analysis. (**A**) Number of RMS reads mapping towards *in silico* generated RMS clusters from target species. (**B**) Correlation between 'true' and 'estimated' target bacteria concentrations
